# Effects of Physical Activity During Pregnancy on Neonatal Birth Weight

**DOI:** 10.1038/s41598-019-42473-7

**Published:** 2019-04-12

**Authors:** Malshani L. Pathirathna, Kayoko Sekijima, Mieko Sadakata, Naoshi Fujiwara, Yoshiyuki Muramatsu, Kuruppu M. S. Wimalasiri

**Affiliations:** 1 Department of Nursing, Graduate School of Health Sciences, Niigata University, Niigata, 951-8518 Japan; 2 Department of Nursing, Faculty of Allied Health Sciences, University of Peradeniya, Peradeniya, 20400 Sri Lanka; 3 Department of Medical Technology,Graduate School of Health Sciences, Niigata University, Niigata, 951-8518 Japan; 4 Department of Food Science and Technology, Faculty of Agriculture, University of Peradeniya, Peradeniya, 20400 Sri Lanka

## Abstract

We assessed the prevalence of adherence to the American College of Obstetricians and Gynecologists (ACOG) recommendations regarding physical activity during pregnancy among Sri Lankan women and explored the relationship between physical activity during pregnancy and neonatal birth weight. In total, 141 pregnant women (gestational age, 18–24 weeks) were included from October to December 2015 and followed up until delivery. A validated questionnaire regarding physical activity during pregnancy was administered in the second and third trimesters. Activities were grouped by type (household/caregiving, occupational, transportation, sports/exercise, and inactivity) and intensity {sedentary [<1.5 metabolic equivalents (METs)], light intensity [1.5–2.9 METs], moderate intensity [3.0–6.0 METs], and vigorous intensity [>6.0 METs]}. Women were categorised as active or inactive based on the ACOG recommendations. In total, 79.1% and 45.2% of women met the guidelines in the second and third trimesters, respectively. The overall time spent and total energy expenditure was significantly higher in the second trimester (p < 0.001). We found no relationship between physical activity during pregnancy and neonatal birth weight. This study indicates that a considerable reduction of time and total energy expenditure occur as pregnancy progresses. Physical activity during pregnancy does not appear to significantly affect neonatal birth weight.

## Introduction

There is growing evidence that physical activity (PA) during pregnancy is beneficial for both the woman and foetus. The American College of Obstetricians and Gynecologists (ACOG) recommends that pregnant women who are free of medical or obstetrical complications engage in ≥30 minutes of moderate PA per day on most if not all days of the week^1^. Despite this recommendation, the worldwide prevalence of PA during pregnancy is reportedly low because many women choose to reduce their level of PA and increase relaxation during pregnancy^2^. The performance of moderate to vigorous PA in early pregnancy is particularly low among women of South Asian and Middle East origin^3^. The main reasons for reduced levels of PA during pregnancy are physical discomfort, complications associated with pregnancy, growth of the woman’s body, and a sense of insecurity with PA^4^.

In 2003, Davies *et al*.^5^ provided new information on the effects of moderate PA on the pregnant woman and foetus, showing that PA has no adverse effects on either maternal or foetal health. Another study showed evidence of a protective effect of mild PA during pregnancy in the second trimester on low birth weight (LBW), preterm birth, and intrauterine growth restriction^6^. A systematic review and meta-analysis of observational studies reported an inverted U-shaped curve; moderate PA levels were associated with increased birth weight but high levels were associated with decreased birth weight^7^.

Sri Lanka is a developing country in southern Asia, in which LBW (less than 2500 g regardless of gestational age) constitutes a significant public health problem^8^. Several factors have been found to be associated with LBW of neonates. Some of these factors include pregnancy-induced hypertension^9^, underweight pre-pregnancy body mass index (BMI) and inadequate gestational weight gain^10,11^, maternal malnutrition^12,13^, active or passive tobacco smoking^14–16^ and exposure to biomass fuel smoke during cooking^17,18^, and anaemia^19,20^.

The effect of PA during pregnancy on pregnancy outcomes was recently established as a research interest. The districts in Sri Lanka with a LBW prevalence of >18% are predominantly those where >40% of the population is engaged in agriculture, and >30% of these individuals are women^21,22^. These findings may reflect the negative birth outcomes associated with increased PA during pregnancy. The prevalence of adherence to the ACOG guidelines regarding PA among pregnant women in Sri Lanka remains unknown. However, the culturally adopted pattern of PA during pregnancy among Sri Lankans is characterised by avoidance of strenuous activities, heavy lifting, exercise, and even fast walking. To our knowledge, the relationship between PA during pregnancy and neonatal birth weight has not been explored specifically in Sri Lankan women. Considering the unknown impact of PA on LBW in Sri Lankan context, this prospective longitudinal study was performed to evaluate patterns of PA during pregnancy and the relationship between PA and neonatal birth weight.

## Methods

### Design, setting, and participants

The present study was part of a larger study designed to assess the maternal factors associated with neonatal birth weight. The study was conducted in a tertiary-care hospital in Sri Lanka during the 9-month period from October 2015 to June 2016. A detailed description of the patient recruitment procedure has been published elsewhere^23^. Initially, 150 pregnant women at a gestational age of 18 to 24 weeks were included in the study and they were followed-up until the delivery. Women with multiple foetuses, a history of miscarriage/abortion, pregnancy-induced hypertension, gestational diabetes mellitus, and chronic medical diseases (e.g., psychiatric disorders; cardiac, renal, or lung disease) were excluded at the time of recruitment, or whenever such a condition was identified during pregnancy. Neonates with a low Apgar score of <5 at 5 minutes after birth were also excluded from the final sample.

### Ethical considerations

The study was approved by the ethical review committees of the Graduate School of Health Sciences, Niigata University, Japan (No. 125); Faculty of Allied Health Sciences, University of Peradeniya; and the Institutional Ethical Review Committee of the Teaching Hospital, Kurunegala, Sri Lanka (ERC/2015/06). It was performed in compliance with the ethical principles outlined in the Declaration of Helsinki. Furthermore, prior to data collection, informed written consent was obtained from all participants.

### Procedure

Socio-economic and demographic data were collected using a pre-designed and pre-tested interviewer-administered questionnaire. Maternal height and weight were recorded by a trained research assistant. Height was measured at the time of recruitment, whereas body weight was measured using a Tanita Digital Scale at each clinic visit prior to delivery. Body weight measurements taken prior to recruitment and at the time of delivery were directly obtained from pregnancy cards and hospital records respectively. Maternal PA was assessed once during the second trimester (approximately 22 weeks of gestation) and once during the third trimester (approximately 34weeks of gestation) using a validated pregnancy PA questionnaire (PPAQ)^24^. The PPAQ was a semi-quantitative questionnaire that asked participants to report the average time spent in 33 activities (31 multiple-choice plus 2 open-ended questions). The women were asked to choose the best approximate amount of time spent in each activity per day or week during the on-going trimester. The provided time durations ranged from 0 to ≥6 hours per day and from 0 to ≥3 hours per week. Self-administration of the PPAQ took approximately 7 to 10 minutes. The total gestational weight gain and neonatal data were collected from the hospital records after all participating women had delivered their babies.

### Calculation of total energy expenditure

Each activity was assigned an intensity using the metabolic equivalent (MET) table^25^. The MET is a unit used to estimate the metabolic cost of PA. The value of 1− MET is approximately equal to a person’s resting energy expenditure. The self-reported time spent in each activity was then multiplied by the corresponding intensity to obtain the average energy expenditure per week (MET-hours/week). When the reported time was in hours per day, it was multiplied by 7 to determine the average weekly energy expenditure. Activities were classified into 5 categories by type: household/caregiving (13 activities), occupational (5 activities), transportation (3 activities), sports/exercises (7 activities plus 2 open-ended questions), and inactivity (3 activities). In addition, each activity was classified into 4 categories based on its intensity: sedentary (<1.5 METs), lightintensity (1.5–2.9 METs), moderate intensity (3.0–6.0 METs), and vigorous intensity (>6.0 METs). The women were categorised into two groups based on the ACOG recommendations as active (≥30 min of moderate PA per day) and inactive (<30 min of moderate PA per day). The women were also categorised into four groups: those who stayed active (active at both 22 and 34 weeks), stayed inactive (inactive at both 22 and 34 weeks), became active (inactive at 22 weeks and active at 34 weeks), and became inactive (active at 22 weeks and inactive at 34 weeks). The outcome variables were the total number of hours per week and total number of MET hours per week.

### Statistical analysis

All statistical analyses were performed using Minitab version 17 (Minitab Inc., State College, PA, USA). Mean with standard deviation (SD) of time spent in PA (hours/week) and energy expenditure (MET-hours/week) are reported for 22 and 34 weeks of gestation separately. Categorical data are expressed as percentages. All variables were first assessed using numerical and graphical techniques to determine whether they met the distributional assumptions of the statistical tests used to analyse them. The Anderson-Darling test was used to explore whether outcome variables were normally distributed; and it was used with the normal probability plot. Participants’ characteristics were compared between active and inactive women using one-way analysis of variance (ANOVA) and the paired t-test. The levels of PA at 22 and 34 weeks were compared using a paired t-test for each type and intensity of PA. Pregnancy outcomes were first assessed based on the categories in the ACOG recommendations using a two-sample t-test and based on the change in the pattern of PA during pregnancy using one-way ANOVA. Neonatal birth weight was first adjusted for the gestational age and thereafter a regression analysis was used to estimate the effect of PA during pregnancy second trimester (active/inactive) on adjusted neonatal birth weight. The covariates in the initial model were maternal age, pre-pregnancy BMI, and gestational age, while the factors were PA during pregnancy (1 = active, 2 = inactive), level of education (1 = none/up to primary, 2 = secondary/higher), monthly household income [1 = up to 13,999 Sri Lankan rupee (LKR), 2 = 14,000–31,999 LKR, 3 = ≥32,000 LKR], area of residence (1 = urban, 2 = suburban, 3 = rural), history of LBW (1 = yes, 2 = no), and parity (1 = primiparous, 2 = multiparous). Variables with the highest p value were removed from the initial model one variable at a time until the final model contained only the variables with a p value of <0.1. A p value of <0.05 was considered statistically significant.

## Results

In total, 150 patients were recruited into the study. Nine women were excluded from the study because of spontaneous abortions (n = 2), multiple foetuses identified by 20 weeks using ultrasound (n = 2), maternal desire to deliver at another hospital (n = 3), and personal withdrawal (n = 2). Of the remaining 141 patients, data regarding total gestational weight were missing for 22 patients. The researcher failed to find neonatal data for 14 neonates from the hospital delivery registry records because of confusion due to many similar names, and one neonate with a 5-minute Apgar score of <5 was excluded at the end of the study, resulted for 126 neonatal data set.

### Participants’ characteristics

The overall background characteristics of the participants have been published separately^23^. Final sample consisted of 126 healthy new-borns and among them 22(17.4%) were LBW babies. Among the 141 women, only 139 (98.6%) at 22 weeks of gestation and 62 (44.0%) at 34 weeks of gestation responded to the PPAQ. At 22 weeks of gestation, 110 (79.1%) women were above the ACOG guidelines regarding PA during pregnancy, whereas only 28 (45.2%) women met the guidelines at 34 weeks of gestation. Table 1 shows the level of moderate PA in relation to maternal and neonatal characteristics. No difference in the women’s characteristics was found based on the moderate PA level.

**Table 1:** Participants’ characteristics based on level of moderate physical activity.

	22 weeks gestation			34 weeks gestation		
	Moderate-intensity physical activity		P Value	Moderate-intensity physical activity		P Value
	<30 min/day (Inactive) n (%)	≥30 min/day (Active) n (%)		<30 min/day (Inactive) n (%)	≥ 30 min/day (Active) n (%)	
All	29 (20.9)	110 (79.1)		34 (54.8)	28 (45.2)	
**Level of education**						
No/Up to primary	3 (13.6)	19 (86.4)	0.325	3 (37.5)	5 (62.5)	0.335
Secondary/Higher	26 (22.6)	89 (77.4)		29 (55.8)	23 (44.2)	
**Monthly household income**						
Up to 13999 LKR	2 (7.1)	26 (92.9%)	0.078	25 (58.1)	18 (41.9)	0.640
14000–31999 LKR	21 (23.1)	70 (76.9)		5 (50.0)	5 (50.0)	
≥32000 LKR	6 (33.3)	12 (66.7)		—	—	
**Area of residence**						
Urban	3 (23.1)	10 (76.9)	0.340	1 (25.0)	3 (75.0)	0.384
Sub-urban	16 (26.7)	44 (73.3)		17 (60.7)	11 (39.3)	
Rural	10 (15.9)	53 (84.1)		14 (51.2)	13 (48.1)	
**History of LBW deliveries**						
Yes	5 (17.9)	23 (82.1)	0.661	5 (35.7)	9 (64.3)	0.116
No	24 (21.6)	87 (78.4)		28 (59.6)	19 (40.4)	
**History of miscarriage/abortions**						
Yes	5 (13.2)	33 (86.8)	0.170	10 (62.5)	6 (37.5)	0.432
No	24 (23.8)	77 (76.2)		23 (51.1)	22 (48.9)	
**Parity**						
Primiparous	11 (24.4)	34 (75.6)	0.472	9 (64.3)	5 (35.7)	0.384
Multiparous	18 (19.1)	76 (80.8)		24 (51.1)	23 (48.9)	
**Pre-pregnancy BMI category** ^**a**^						
Underweight	9 (31.0)	20 (69.0)	0.282	6 (60.0)	4 (40.0)	—
Normal	12 (15.2)	67 (84.8)		22 (59.5)	15 (40.5)	
Overweight	6 (26.1)	17 (73.9)		5 (41.7)	7 (58.3)	
Obese	2 (25.0)	6 (75.0)		—	2 (100)	
**Total gestational weight gain category** ^**b**^						
Within recommended	5 (14.3)	30 (85.7)	0.543	9 (60.0)	6 (40.0)	0.693
Below recommended	18 (23.4)	59 (76.6)		20 (54.1)	17 (45.9)	
Over recommended	1 (20.0)	4 (80.0)		1 (33.1)	2 (66.7)	
**Neonatal birth weight category**						
<2500 g	2 (19.5)	19 (90.5)	0.100	5 (50.0)	5 (50.0)	0.640
≥2500	27 (26.2)	76 (73.8		25 (58.1)	18 (41.9)	
**Mode of delivery**						
Normal delivery	18 (23.1)	60 (76.9)	0.915	23 (65.7)	12 (34.3)	0.062
Cesarean section	11 (23.9)	35 (76.1)		7 (38.9)	11 (61.1)	

^a^Based on World Health Organization international BMI cut-off values^40^, ^b^Based on Institute of Medicine (IOM) 2009 re-examined guidelines^37^.

### Comparison of PA level during pregnancy between the second and third trimesters

The number of hours spent for all modes of activity per week was significantly higher in the second than third trimester (p < 0.001). When the activities were divided into groups according to their intensity, a significant reduction in time spent in PA during the third trimester compared with the second trimester was observed in terms of sedentary activities (p < 0.05), light intensity (p < 0.001), and moderateintensity (p < 0.05). The mean time spent in vigorous-intensity activity was very low in both the second and third trimesters, and the difference did not reach statistical significance. A similar pattern was observed for the total energy expenditure (MET-hours/week) except for sedentary activities. The average energy expenditure corresponding to sedentary activities was significantly higher in the third than second trimester (p < 0.001). Division of the activities by type revealed significant reduction of time (hours/week) spent in household/caregiving activities (p < 0.01), transportation (p < 0.001), and inactivity (p < 0.05) during the third trimester (Table 2). A similar pattern was observed for the total energy expenditure.

**Table 2:** Comparison of physical activity levels in second and third trimesters (n = 62).

	h/week		Difference (95% CI for the difference)	P value	MET h/week		Difference (SD) [95% CI for the difference]	P value
	22 weeks	34 weeks			22 weeks	34 weeks		
	Mean (SD)	Mean (SD)			Mean (SD)	Mean (SD)		
**All modes of activities**	11.9 (5.0)	7.7 (3.8)	4.2 (6.3) [2.6−5.8]	<0.001*	145.8 (67.2)	78.1 (41.7)	67.7 (80.0) [47.4−88]	<0.001*
**By intensity**								
Sedentary	3.6 (3.1)	2.7 (2.3)	0.9 (3.2) [0.1−1.7]	<0.05*	26.4 (22.4)	46.0 (28.7)	−19.6(39.0) [−29.5–9.7]	<0.001*
Light-intensity	5.3 (2.0)	3.1 (1.8)	2.2 (2.8) [1.5−2.9]	<0.001*	81.6 (30.8)	19.6 (16.6)	62.0 (36.0) [52.9−71.2]	<0.001*
Moderate-intensity	2.9 (2.4)	1.7 (1.4)	1.2 (2.5) [0.5−1.8]	<0.001*	37.2 (38.3)	13.6 (13.0)	23.6 (38.2) [13.9−33.3]	<0.001*
Vigorous-intensity	0.1 (0.4)	0.0 (0.0)	0.1 (0.4) [−0.0–0.2]	0.131	0.5 (2.8)	0.0 (0.0)	0.5 (2.8) [−0.2−1.3]	0.132
**By type**								
Household/care giving	4.3 (2.5)	3.3 (1.8)	1.1 (3.0)[0.4−1.8]	<0.01*	99.2 (55.8)	49.9 (31.0)	49.3 (62.1)[33.6−65.1]	<0.001*
Occupational	0.3 (1.3)	0.1 (0.8)	0.2 (1.1)[−0.0−0.5]	0.096	3.9 (15.3)	1.1 (8.5)	2.8 (13.0)[−0.46−6.1]	0.090
Sports/exercise	1.6 (1.7)	1.3 (1.0)	0.3 (1.8) [−0.1−0.8]	0.169	5.8 (6.4)	4.6 (3.4)	1.2 (6.9) [−0.6−2.9]	0.180
Transportation	0.6 (0.6)	0.2 (0.4)	0.4 (0.8) [0.2−0.6]	<0.001*	8.9 (9.4)	2.9 (5.5)	6.0 (11.2) [3.1−8.9]	<0.001*
Inactivity	3.8 (3.1)	2.7 (2.3)	1.0 (3.2) [0.2−1.8]	<0.05*	28.0 (22.9)	19.6 (16.6)	8.3 (23.6) [2.3−14.3]	<0.01*

Comparisons were performed using the paired-t-test. *p < 0.05, CI: confidence interval.

### Level of PA in relation to changing pattern of PA

Table 3 compares the PA levels during the second and third trimesters among the groups of women based on their changing patterns of PA during pregnancy. The average time spent per week in PA was significantly lower during the third than second trimester in the “became inactive group” (p < 0.001). A similar pattern was observed for the total energy expenditure during the third trimester in the “became inactive” group of women (p < 0.001). Women in the “stayed active” and “stayed inactive” groups also showed a lower total energy expenditure in the third than second trimester (p < 0.05). Both the time spent per week and average energy expenditure per week in the third trimester were significantly higher in women who “stayed active” throughout the pregnancy than in women who “stayed inactive” and “became inactive” (p < 0.001).

**Table 3:** Physical activity levels in relation to changing patterns of physical activity during pregnancy (n = 62).

	n (%)	Time spent for PA (hours/week)				Energy expenditure (MET-hours/week)			
		22 weeks gestation	P value	34 weeks gestation	P value	22 weeks gestation	P value	34 weeks gestation	P value
Stayed active	25 (41.0)	11.8 (5.1)	0.188^‡^	10.0 (4.2)^a^	<0.001^‡^* 0.208^†^ 0.054^††^ 0.602^†††^ <0.001^††††^**	151.6 (73.0)	0.425^‡^	110.4 (41.4)^a^	<0.001^‡^* 0.046^†^* <0.010^††^* 0.329^†††^ <0.001^††††^*
Stayed inactive	7 (11.5)	8.3 (3.0)		5.6 (3.1)^b,c^		108.2 (37.5)		48.7 (33.5)^b,c^	
Became active	3 (4.9)	11.0 (5.5)		8.5(1.5)^a,b,c^		127.1 (56.3)		84.3 (7.1)^a,b,c^	
Became inactive	26 (42.6)	13.0 (5.2)		5.9 (2.3)^c^		152.8 (69.2)		54.8 (21.6)^c^	

P value refers to that obtained by the paired t-test unless otherwise stated. ^‡^One-way analysis of variance. Comparison of physical activity at 22 and 34 weeks of gestation in the ^†^active group, ^††^inactive group, ^†††^became active group, and ^††††^became inactive group. ^a,b,c^Values with the same superscript lowercase letter do not represent a significance difference. *p < 0.05.

### Changing pattern of PA during pregnancy and pregnancy outcomes

No difference was observed in the total gestational weight gain, gestational age, or neonatal birth weight based on either the ACOG recommendations or the changing pattern of PA during pregnancy (p > 0.05) (Table 4).

**Table 4:** Pregnancy outcomes in relation to physical activity during pregnancy.

		n (%)	Total gestational weight gain (kg) Mean (SD)	P Value	Gestational age (weeks) Mean (SD)	P Value	Neonatal birth weight (g) Mean (SD)	P Value
ACOG recommendations of pregnancy PA	Active at 22 weeks	95 (76.6)^a^	9.5 (3.7)	0.530	38.9 (1.4)	0.506	2853.0 (491)	0.330
	Inactive at 22 weeks	29 (23.4)^a^	8.9 (3.9)		38.6 (1.6)		2961.0 (526)	
	Active at 34 weeks	23 (43.4)^a^	9.3 (3.1)	0.636	38.8 (1.3)	0.154	2953.0 (572)	0.915
	Inactive at 34 weeks	30 (56.6)^a^	9.7 (2.8)		39.3 (0.9)		2962.0 (516)	
Changing pattern of pregnancy PA	Active	20 (37.7)	9.7 (2.9)	0.341^‡^	39.0 (1.0)	0.097^‡^	2981.0 (587.8)	0.431^‡^
	Inactive	7 (13.2)	9.9 (1.9)		39.0 (1.1)		3235.7 (573.5)	
	Became active	3 (5.7)	6.5 (3.9)		37.7 (2.6)		2776.7 (500.8)	
	Became inactive	23 (43.4)	9.7 (3.1)		39.4 (0.9)		2878.3 (479.3)	

Pvalue refers to that obtained by the two-sample t-test unless otherwise stated. ^‡^One-way analysis of variance. ^a^n values correspond to the gestational age and neonatal birth weight.

### Effects of PA level during pregnancy on neonatal birth weight

Assessment using the regression analysis was limited to the PA level during the second trimester because of the low PPAQ response rate during the third trimester. The regression analysis revealed significant impacts of gestational age on neonatal birth weight (p < 0.05). We found no significant impact of moderate PA during the second trimester on neonatal birth weight (p = 0.718) (Table 5).

**Table 5:** Regression analysis for adjusted mean birth weight (g), n = 122; R^2^ (adjusted) = 63.99%.

Term	Coefficient	95% CI	Contribution	t value	P value
Constant	−1190	−1775–−604		−4.03	<0.001*
Pre-pregnancy BMI	−1.0	−5.9–3.8	1.39%	−0.43	0.671
Gestational age	105.0	90.6–119.4	64.10%	14.45	<0.001*
**Second trimester activity level (active- reference level)**					
Second trimester activity level (inactive)	8.9	−40.1–57.9	0.01%	0.36	0.718
**Area of residence (urban-reference level)**					
Area of residence (sub-urban)	4.3	−66.8–75.5	0.56%	0.01	0.904
Area of residence (rural)	31.5	−39.6–102.7		0.88	0.382
**Previous history of LBW (yes- reference level)**					
Previous history of LBW (no)	1.6	−54.9–58.2	0.01%	0.06	0.954
**Parity (primiparous-reference level)**					
Parity (multiparous)	4.8	−42.7–52.3	0.01%	0.20	0.842

*p < 0.05.

## Discussion

Of all participants, 98.6% responded at 22 weeks of gestation, but only 44% responded at 34 weeks of gestation, reflecting loss to follow-up. In Sri Lanka, maternal care is free throughout the ante-, intra- and postnatal periods. Antenatal care is provided by maternal and child health clinics, both in the field and in medical institutions. Most of the women in the current study attended both types of clinics. As institutional clinics are usually very crowded, the women sometimes tended to attend only field clinics, which do not perform advanced procedures such as blood evaluations or ultrasound on women with low-risk pregnancies at 34 weeks of gestation. In total, 79.1% and 45.2% of the pregnant women who participated in this study met the ACOG recommendations for PA during pregnancy in the second and third trimesters, respectively. This rate is significantly higher than the rates recorded in China^26^ (11.1%), Ireland^27^ (21.5%), and the United States^28^ (22.9%). However, the average energy expenditure in the second trimester among the women in the current study was lower than that reported in previous studies in Australia^29^ and France^30^. Moreover, in the current study, there was a significant reduction in the time spent in PA and the total energy expenditure during the third trimester. In addition to physical discomfort and bodily growth, this reduction may be related to the traditional Sri Lankan culture, which defines pregnancy as a vulnerable period that requires rest and protection. Therefore, pregnant Sri Lankan women tend to obey the traditional beliefs of no lifting heavy objects, no fast walking, and no strenuous activities during pregnancy. As a result, vigorous activity during the second trimester was very low in the present study, and no women reported vigorous activities during the third trimester. In addition, nearly half of the total energy expenditure during the third trimester was covered by sedentary activities, highlighting the increase in low activity as pregnancy progresses. A major proportion of time spent in PA and the total energy expenditure among the pregnant women in the current study was covered by household/caregiving activities.

In 2017, Bisson *et al*.^31^ found that pregnant women engaging in high-level sports/ exercise during the first trimester delivered infants of lower birth weight. Similarly, a cohort study performed in Iran reported that sporting activities increased the risk of an LBW infant^32^. We found that most Sri Lankan women did not engage in sport/exercise, rendering comparisons difficult. This is a common feature of many Asian countries; women of reproductive age carry the most substantial burden of the household and caregiving activities. Although it did not reach statistical significance, most women in the lowest income group (92.9%) were active during the second trimester, indicating an association between household income and energy expenditure in Sri Lanka. The analysis of the changing pattern of PA during pregnancy revealed that 41% of women stayed active throughout the pregnancy while 42.6% women became inactive during the third trimester.

Our univariate analyses showed no relationships between PA during pregnancy and gestational weight gain, gestational age, and neonatal birth weight. These findings are in agreement with the results of previous studies^33,34^, which indicated no significant association between maternal PA and gestational weight gain, gestational age, or neonatal birth weight. In contrast, some studies have shown a protective effect of mild PA against LBW and preterm birth^6^, whereas others have shown an increased risk of intrauterine growth restriction with moderate or vigorous occupational PA^35^.

In 2003, Cohen *et al*.^36^ indicated that pregnant women who engaged in purposeful walking routines associated with activities of daily living achieved healthy weekly weight gains. However, in the current study, we did not focus on weekly gestational weight gains or the relationships between various PAs and birth outcomes. Moreover, Cohen *et al*.^36^ showed that moderate-intensity sports/exercise was not associated with appropriate gestational weight gain^37^ and stressed that sedentary pregnant women should not be encouraged to engage in vigorous activity to achieve the recommended weight gain^37^. However, Cohen *et al*. focused on prevention of overweight; most enrolled subjects exceeded the recommended gestational weight gains^37^. Therefore, the meaning of “appropriate weight gain” in the cited work may differ from ours; most of the women we enrolled exhibited less weight gain than the recommended total gestational weight gain. The resultsof the current study showed that women who were below the ACOG recommendations of PA during pregnancy delivered neonates with a higher mean birth weight than did women who did not meet the ACOG recommendations, although the difference was not statistically significant. On the contrary, a systematic review^7^ suggested that moderate PA during pregnancy was associated with increased birth weight, but high PA was associated with reduced birth weight. However, these associations remained when leisure time PA alone was considered. Moreover, more than one-third of all studies included in the cited review were not adjusted for covariates known or thought to influence both PA and foetal growth^7^. The initial sample size of our current study was chosen to afford a power of 0.8 at an alpha level of 0.05. However, the loss to follow-up experienced at 34 weeks of gestation reduced the statistical power of our tests and rendered it difficult to draw robust conclusions; our data lost at least some significance. One hypothesis states that an inverse relationship exists between PA during pregnancy and utero-placental blood flow, ultimately leading to a reduced foetal oxygen and glucose supply. However, the proportion of women who engaged in vigorous PA in our study was very low, rending it difficult to draw robust conclusions on any association between increased PA and birth weight. In addition, our sample size was likely insufficient to detect the significance of such a relationship. Therefore, the results regarding observed association between PA and Neonatal birth weight may require further inquiry and verification before firm conclusion can be reached. Although no relationship between PA during pregnancy and pregnancy outcomes was observed in this group of women, the associations between postpartum weight retention and other medical/obstetric complications were not assessed. Large-scale studies are warranted to assess the factors influencing PA during pregnancy and the effects of PA during pregnancy on the mother and infant even after childbirth. The findings regarding PA during the second trimester and neonatal birth weight need to be confirmed in further large-scale studies.

The strengths of our study included the use of a reliable questionnaire affording reasonably accurate measures of a variety of PAs performed during pregnancy. This is the first validated questionnaire that can be employed to determine the lower and upper extent of PA, in terms of frequency, duration, and intensity, during pregnancy^24^. The fact that the PPAQ is brief and user-friendly renders it attractive to researchers who wish to examine changes in PA during pregnancy, and also to prenatal healthcare providers who wish to address PA in pregnant women^24,38,39^. Despite these strengths, our study had certain limitations. First, although we used a validated and reliable instrument, the reliability of our data may have been affected by recall bias and the cognitive burden associated with instrument self-administration. Many women were lost to follow-up; the PPAQ response rate was very low in the third trimester, rendering the sample size much smaller than expected. Also, we did not evaluate the use of medications during pregnancy, intra-uterine infection status, placental abnormalities, or genetic factors possibly associated with LBW. Another limitation of this study is that administration of the PPAQ once at around 22 and 34 weeks of gestation may not be a reliable measure of PA during the second and third trimesters.

## Conclusions

More than half of women achieved the recommended PA level during the second trimester of pregnancy, whereas only 45.2% achieved the recommended PA level during the third trimester. Significant reductions in the time spent in PA and the total energy expenditure were observed as pregnancy progressed. No significant association between PA during pregnancy and neonatal birth weight was found.
